# Understanding the evolution of a de novo molecule generator via characteristic functional group monitoring

**DOI:** 10.1080/14686996.2022.2075240

**Published:** 2022-06-01

**Authors:** Takehiro Fujita, Kei Terayama, Masato Sumita, Ryo Tamura, Yasuyuki Nakamura, Masanobu Naito, Koji Tsuda

**Affiliations:** a Polymer Design Group, Research and Services Division of Materials Data and Integrated System (MaDIS), National Institute for Materials Science (NIMS)Data-driven, Tsukuba, Japan; b Graduate School of Medical Life Science, Yokohama City University, Tsurumi-ku, Japan; c RIKEN Center for Advanced Intelligence Project, RIKEN Center for Advanced Intelligence Project, Tokyo, Japan; d International Center for Materials Nanoarchitectonics (WPI-MANA), National Institute for Materials Science (NIMS), Tsukuba, Japan; e Research and Services Division of Materials Data and Integrated System, National Institute for Materials Science (NIMS), Tsukuba, Japan; f Graduate School of Frontier Sciences, The University of Tokyo, Kashiwa, Japan

**Keywords:** characteristic functional group monitoring, chromophore, De novo molecule generation, deep learning

## Abstract

Recently, artificial intelligence (AI)-enabled de novo molecular generators (DNMGs) have automated molecular design based on data-driven or simulation-based property estimates. In some domains like the game of Go where AI surpassed human intelligence, humans are trying to learn from AI about the best strategy of the game. To understand DNMG’s strategy of molecule optimization, we propose an algorithm called characteristic functional group monitoring (CFGM). Given a time series of generated molecules, CFGM monitors statistically enriched functional groups in comparison to the training data. In the task of absorption wavelength maximization of pure organic molecules (consisting of H, C, N, and O), we successfully identified a strategic change from diketone and aniline derivatives to quinone derivatives. In addition, CFGM led us to a hypothesis that 1,2-quinone is an unconventional chromophore, which was verified with chemical synthesis. This study shows the possibility that human experts can learn from DNMGs to expand their ability to discover functional molecules.

## GRAPHICAL ABSTRACT

**Figure:**
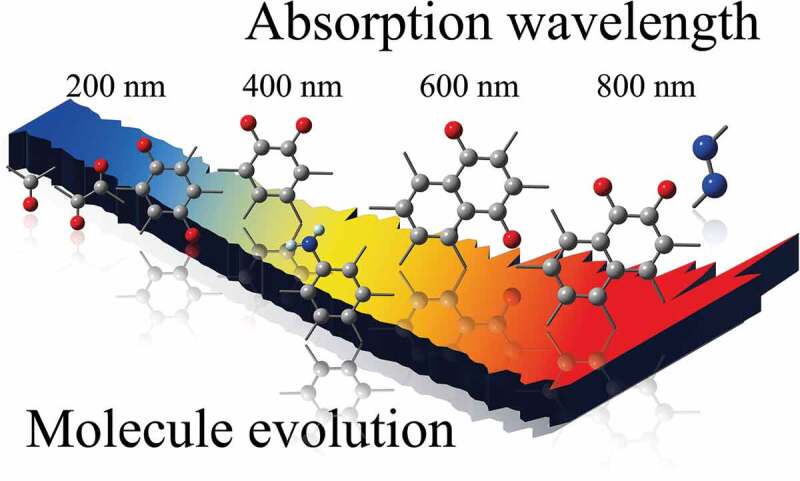

## 1. Introduction

Recently, de novo molecular generators (DNMGs) that combine machine learning and quantum chemical calculation have been intensively developed [1–6]; these approaches have led to the successful production of molecules as good as or better than those imaged by human under some circumstances [1,7,8]. In the fields where AI surpassed human intelligence such as Shogi and the game of Go, humans are trying to learn from AI to enhance their abilities. In Shogi [9], young players are actively using AI-shogi systems to develop their skills. Shin et al. [10] reported that, after AlphaGo [11], human Go players are more likely to take AI-like decisions. A key question here is if chemists can learn from AI-based systems such as DNMGs and improve their skills in developing functional compounds. Unfortunately, current DNMGs are almost indiscernible and not suitable for skill acquisition by chemists.

In this paper, we develop a method to distill useful knowledge from molecules generated by a DNMG. Since a DNMG continuously learns from an evaluator based on quantum chemical calculation, it gets ‘cleverer’ as time passes. We are interested in strategic evolution of a DNMG from a quasi-random strategy to more informed and targeted one. Given a time series of generated molecules, our method termed *characteristic functional group monitoring* (CFGM) finds statistically enriched functional groups in a sliding window. For a functional group, its occurrence probability in the generated molecules is compared to that in the training set. If the ratio of probabilities (i.e. odds ratio) is high, we regard that the functional group is focused on by the DNMG. Chemists, in turn, may be able to infer why the functional group works by means of additional experiments and simulations.

Our approach is tested in the task of generating molecules that absorb light of long wavelengths. Applications of such molecules include organic light emitting diodes [12], dyes [13] and solar cells [14]. From the viewpoint of the molecular electronic structure theory based on quantum mechanics [15], light absorption is mediated by the transition among quantised electronic structures in molecules. In principle, the light absorption of a molecule can be tuned by changing the gap between the highest occupied molecular orbital (HOMO) and the lowest unoccupied molecular orbital (LUMO). Nonetheless, the relationship between light absorption and molecular structure is not fully understood; for example, it is still challenging to explore the limitation of absorption wavelength of small molecules [16]. Conventionally, photofunctional molecules are often designed using a known *chromophore* [17], i.e. the part of a molecule responsible for its color. The total number of known organic chromophores is unclear, but a reasonable bottom line would be obtained from the chromophore database by Joung et al. [17] that contains 7,016 chromophores.

Our method was applied to more than 40,000 molecules generated by ChemTS [8], a DNMG that combines reinforcement learning with deep learning, in the task of elongating the absorption wavelength of light, as evaluated by density functional theory (DFT) calculations. In the early stage of generation, diketone and aniline derivatives were highly enriched, but gradually quinone derivatives became more dominant. Among them, derivatives of 1,4-quinon and 1,2-quinon were outstanding. While the former is one of the most well-known chromophores [18], the latter has not been recognized as a chromophore. In fact, the chromophore database [17] contains the former, but not the latter. We validated 1,2-quinon’s function as a potential chromophore by synthesizing a molecule including it and measuring its absorption spectrum. Though more validation experiments would be necessary to establish 1,2-quinon as a new chromophore, our result suggests that CFGM is useful in understanding a DNMG result and can guide chemists to a new pathway of research.

## 2. Methods

### 2.1. Characteristic functional group monitoring

A DNMG uses a *reward function* of molecules that typically depends on quantum chemical calculation. The generated molecules are gradually optimized towards higher reward. CFGM aims to track a set of functional groups that plays main roles in reward optimization. CFGM is applicable to DNMGs that have an *unlabelled* training set, i.e. a set of molecules without their rewards. Such methods include variational-autoencoder-based molecule generators [6], ReLeaSE [4] and ChemTS [2]. Genetic algorithm-based generators [19] are not covered, because they do not need a training set. Most generators using generative adversarial networks (GANs) [5] require a labelled training set, hence CFGM cannot be applied.

In the time series of generated molecules, we consider a sliding window of a predetermined length (for example, 100 molecules). Given a functional group f, the fraction of in-window molecules including it is described as $P\left(f\right)$. In addition, let $P_{t}\left(f\right)$ denote the fraction in the training set. The odds ratio is described as

$$P_{E}\left(f\right)=P\left(f\right)\;/\;P_{t}\left(f\right).$$

A high value of P_E_ indicates that the DNMG has learned to use the functional group to raise the reward. CFGM computes the odds ratios for a set of functional groups to identify which ones are enriched at the current time point. By observing the odds ratios across the entire series of generated molecules, the strategic evolution of a DNMG can be investigated.

### 2.2. ChemTS settings

To elongate the absorption wavelength of pure organic molecules consisting of C, N, O, and H atoms, we used ChemTS, which employs the MCTS [20] algorithm and the recurrent neural network (RNN) [21]. For each generated molecule, its Cartesian coordinates were obtained using the RDKit package [22]. The absorption wavelength was computed using TD-DFT at the B3LYP/3-21 G* level, implemented in the Gaussian 16 package [23]. The lowest 10 states for each molecule were calculated after geometry optimisation. We used the following reward function, *r*(*I*), of a generated molecule, *I*, in the MCTS-based search process:

$$r\left(I\right)=F\left(I\right)*G\left(I\right),$$

$$F\left(I\right)=\frac{\tanh\left(0.003\left(\lambda_{I}-\theta \right)\right)}{2},$$

$$G\left(I\right)=\frac{-\tanh\left(SA_{I}-4\right)+1}{2}.$$

The reward function consists of the product of a term relating to the absorption wavelength, $F\left(I\right)$, and a term relating to synthesizability, $G\left(I\right)$. $F\left(I\right)$takes values between 0 and 1; the longer the computational wavelength, $\lambda_{I}$, of molecule *I*, the larger the value. θ is the comparative criterion, which is set to 400 nm in this study. For a wavelength of 400 nm, the value of $F\left(I\right)$is 0.5. $G\left(I\right)$ also takes values between 0 and 1 and is calculated from the synthetic accessibility score, SA_*I*_, of molecule *I*, which predicts the difficulty of the synthesis.

To accelerate the MCTS search, we adopted the virtual loss strategy [20] to parallelise the computation. We used the following score (*u*_i_) for each child node *i* in the selection step:

$$u_{i}=\frac{tR_{i}}{v_{i}+vl_{i}}+CP_{i}\frac{\sqrt{v_{p}+vl_{p}}}{v_{i}+vl_{i}+1}.$$

Here, $tR_{i}$ is the total reward of node *i*, $v_{i}$ is the total number of visits to node i, i is the total number of virtual visits of i (virtual loss), $vl_{i}$ is the RNN probability of node i, and $v_{p}$ and $vl_{p}$ are the total number of visits and the total number of virtual visits of the parent node, respectively. The parameter C controls the exploration–exploitation trade-off, which is set to 2 in this study. The results for C = 1 and C = 4 showed similar tendencies in CFGM. See Section 2 of Supplementary Information for details.

## 3. Results and discussions

### 3.1. Molecule generation experiment

**Figure 1.**
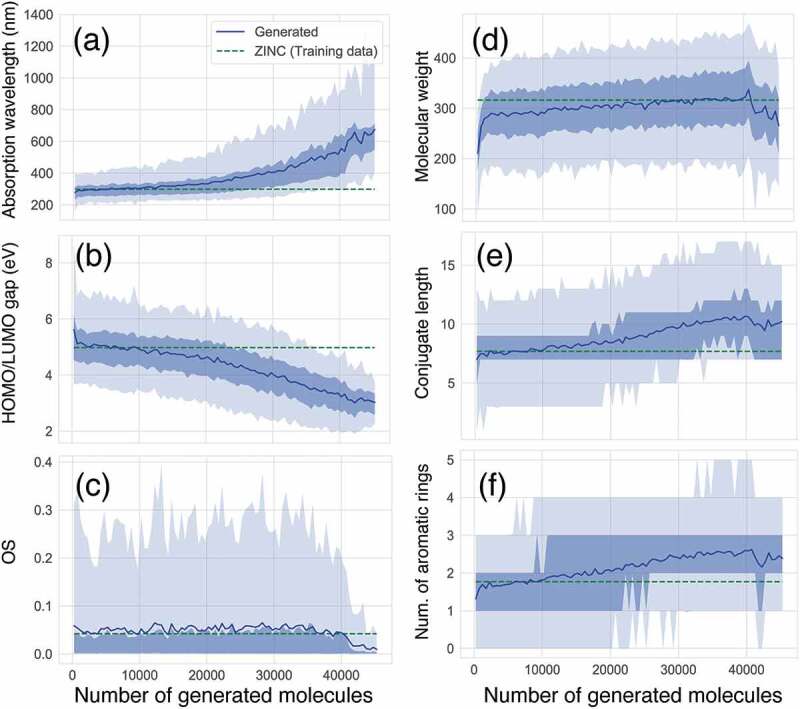
Evolution of molecular properties in the series of generated molecules. (a) absorption wavelength (nm) to S_1_ excited state, (b) HOMO/LUMO gap (eV) (c) absorption intensity (oscillator strength; OS), (d) molecular weight (g mol ^−1^), (e) conjugate length, (f) number of aromatic rings. Average values of training and generated molecules at each step are depicted by green broken line and blue solid line, respectively. The shaded area depicts the distribution profiles of generated molecules for each property. A thin shade area represents 5%–95% of the total distribution, while a dense shade area represents 15%–75% of the total distribution in each number of generated molecules.

We demonstrate how CFGM works with ChemTS in a task of maximising the absorption wavelength. ChemTS was trained with a set of 153,253 SMILES [24] strings that only consisted of H, O, N, and C elements obtained from the ZINC database [25]. It generated 45,321 organic molecules, using 2048 cores over 120 h. See the method section for the reward function and other settings of ChemTS. Before focusing on functional groups, let us investigate the evolution of basic molecular properties. Figure 1(a) shows the evolution of the absorption wavelength. After generating 10,000 molecules, the average value of the absorption wavelength began to elongate and reached more than 600 nm. The maximum absorption wavelength was over 1,200 nm after generating 40,000 molecules. With an increase in the wavelength, the average HOMO/LUMO gap of the generated molecules monotonically decreases, as shown in Figure 1(b). It is known that there is an inversely proportional correlation between the absorption wavelength and the HOMO/LUMO gap. ChemTS also clearly uses this correlation and design molecules whose HOMO/LUMO gap is greater than 1.0 eV. However, the oscillator strength (OS) of the absorption does not grow and saturates around 0.05, which is not different from the average OS of the molecules included in the training data (Figure 1(c)). After 40,000 molecules are generated, the average value of OS is degraded, in contrast to the elongation of the absorption wavelength. This trend agrees with the intuition and statistical results that the organic molecules that absorb the long-wavelength light with high intensity are rare [26]. Hence, the relationship among electronic structures is reasonable. However, the relationship between the electronic structure and the molecular structure is not.

The design principle of ChemTS for molecules that absorb long-wavelength light is not dependent on the expanding molecular size. As shown in Figure 1(d), the average molecular weight gradually increased until 40,000 molecules were generated. However, from the generation of 40,000 molecules, the average molecular weight decreased. This tendency can be observed in the average conjugate length and the number of aromatic rings, as shown in Figure 1(e,f), respectively. Up to the generation of 40,000 molecules, the absorption wavelength increases with increasing conjugation length and the number of aromatic rings (Figure 1(a)). This means that the traditional strategy of longer conjugation lengths leading to longer absorption wavelengths is working well [27], and ChemTS supports the same strategy. However, a slight deviation has occurred since the generation of 40,000 molecules. This means that ChemTS takes another strategy to elongate the absorption wavelength.

### 3.2. Strategic evolution

Table 1 shows the odds ratios (P_E_) of several functional groups with respect to the entire series of generated molecules. Although ketone derivatives are predominantly generated (~50%), their P_E_ value is not high (0.649). Similarly, the P_E_ of traditional chromophore derivatives (azo, aniline) is not high. Hence, ChemTS does not regard the azo and aniline derivatives as suitable molecules for absorbing long-wavelength light. In contrast, 1,4-quinone shows a high P_E_ of >30, despite the low percentage of generated molecules. Among the generated molecules, 1,2-quinone shows a relatively high odds ratio. This result indicates that ChemTS predicted that 1,2-quinone is an important functional group for long-wavelength absorption. Dyes with anthraquinone and 1,4-quinone structures are well known [18]. However, although 1,2-quinone is considered a cofactor [28] and a building block in heterocyclic synthesis [29], it has received little attention as a chromophore. Among the 1,2-quinone derivatives, the P_E_ of 1,2-naphthoquinone is the highest in Table 1, in contrast to 1,4-naphthoquinone, an isomeric derivative of 1,2-naphthoquinone.

**Table 1.** Functional group enrichment analysis for various functional groups and their percentage of generated molecules and training data. Odds ratio is given as P_E._

Functional group	P_E_	Generated mol. (%)	Training data (%)
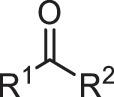 Ketone	0.649	49.7	76.6
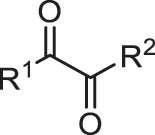 Diketone	0.375	0.847	2.26
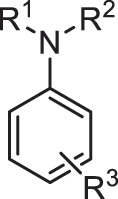 Aniline	0.537	16.2	30.1
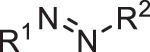 Azo	2.37	0.878	0.371
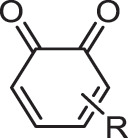 1,2-quinone	15.5	1.05	0.668
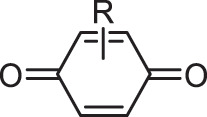 1,4-quinone	31.5	0.682	0.0217
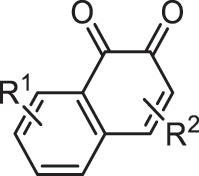 1,2-naphthoquinone	78.5	0.0486	0.000619
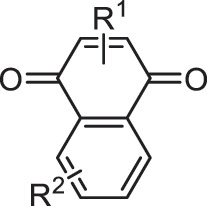 1,4-naphthoquinone	0.938	0.0110	0.0118

**Figure 2.**
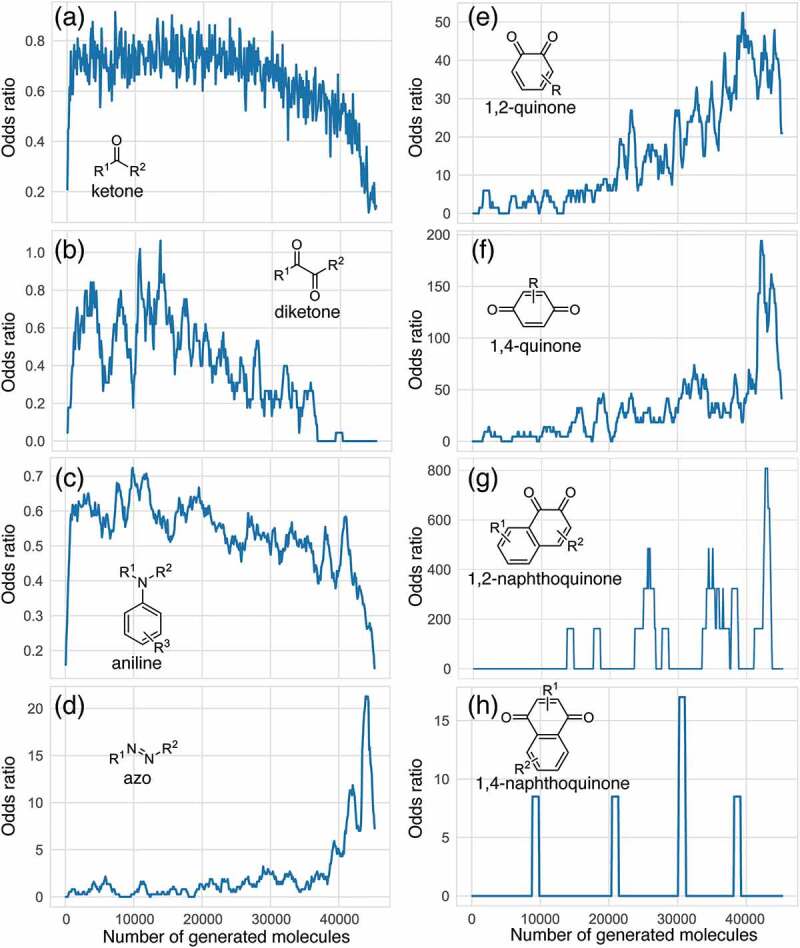
Odds ratio evolution of several functional groups shown in Table 1 as a function of the number of generated molecules.

The evolution of P_E_ for the functional groups shown in Figure 2 indicates that ChemTS changed the strategy from ketones, diketones, and aniline to azo and quinone derivatives. As shown in Figure 2(a–c), ChemTS insisted on ketone, diketone, and aniline derivatives from the initial stage to 20,000 molecule generation. From 20,000, however, P_E_ gradually decreased. Instead, the P_E_ of azo (Figure 2 (d)) and quinone (Figure 2(e–h)) derivatives gradually increased. In particular, the P_E_ of azo, 1,4-quinone, and 1,2-quinone derivatives suddenly increased after the generation of 40,000 molecules. This behaviour corresponds to the elongation of the absorption wavelength, as shown in Figure 1(a). The P_E_ values of 1,4-quinone and 1,2-quinone were considerably higher than that of azo. This indicates that 1,4-quinone is important for designing long-wavelength light absorption chromophores. Additionally, ChemTS predicted the potential of 1,2-quinone derivatives after exploring the possibilities of ketone, diketone, and aniline derivatives.

### 3.3. Experimental validation

**Figure 3.**
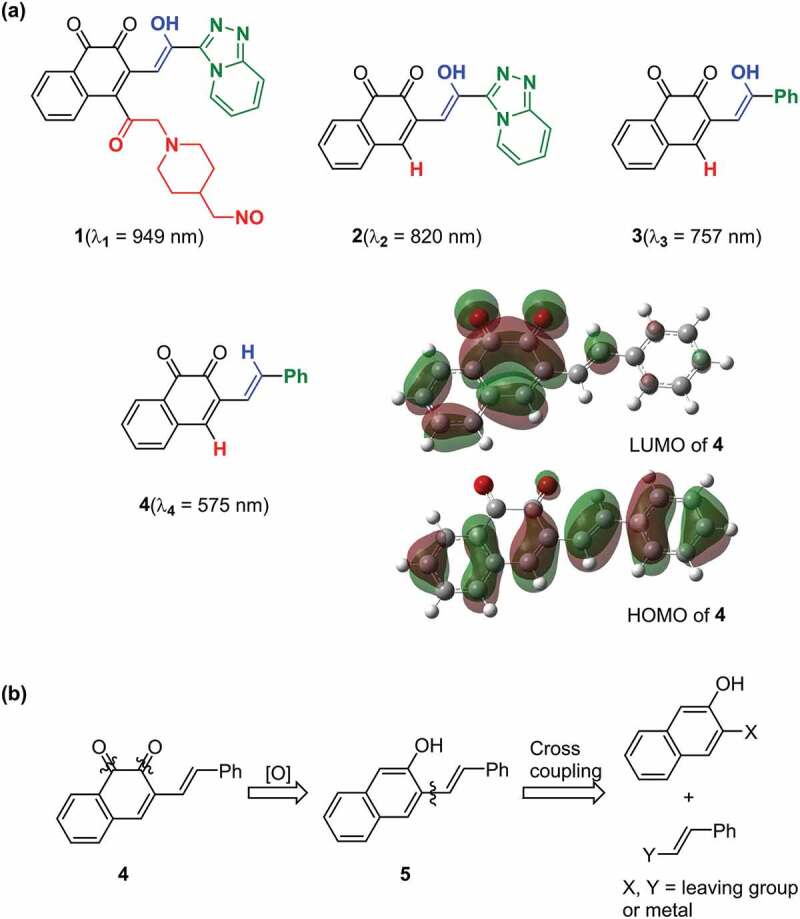
Target molecule inspired by ChemTS. (a) the generated molecule by ChemTS is 1. 2–4 molecules are synthesis models of 1. The absorption wavelength of each molecule is estimated at the APFD/6-31 G* level. Surfaces of HOMO–LUMO orbitals of 4 are drawn at an isodensity value of 0.02. (b) Retro-synthesis of 4.

Among the 1,2-quinone derivatives, 1,2-naphthoquinone shows a high odds ratio of 78.5, and its evolution behaviour (Figure 2(g)) is similar to that of 1,2-quinone, as shown in Figure 2(e). In total, 22 different 1,2-naphthoquinone structures were generated (see Figure S9 of the ESI). Among them, we focused on **1** in Figure 3(a), which showed the longest wavelength absorption of these molecules. Compound **1** has a structure consisting of an enol and a carbonyl skeleton attached to the 1,2-naphthoquinone skeleton; time-dependent DFT (TD-DFT) calculations at the APFD/6-31 G* level (computational level is changed for conformation) predicted the appearance of the absorption at approximately 949 nm (Figure 3(a)). For the actual synthesis, we simplified **1** by trimming the functional groups that would not be as important for their function as chromophores (Figure 3(a)). First, **2** was obtained by replacing the moiety highlighted by red in **1** with hydrogen. According to our preliminary computation, TD-DFT calculations estimated that the absorption wavelength of **2** was approximately 820 nm, which is shorter than that of **1** by 130 nm. Second, **3** was obtained by replacing the triazole group in **2** with the phenyl group that also has an π conjugate structure because the triazole group is difficult to introduce. TD-DFT calculations estimated the absorption wavelength of **3** to be 757 nm. Although enol skeletons are thermodynamically unfavoured and isomerise to their keto forms, we tried synthesising the enol but failed to isolate the desired compound. Accordingly, novel **4**, in which the enol skeleton of **3** was replaced by a simple olefin, was found as a synthesisable model of the target chromophore (**1**). The absorption wavelength of **4** to its first excited state owing to HOMO–LUMO single electron transition is estimated to be 575 nm, which undergoes a considerable blue shift from that of **1**. The nature of excitation to their first excited state is preserved (see Figure S10 of ESI) during the conversion of **1** to **4**.

**Figure 4.**
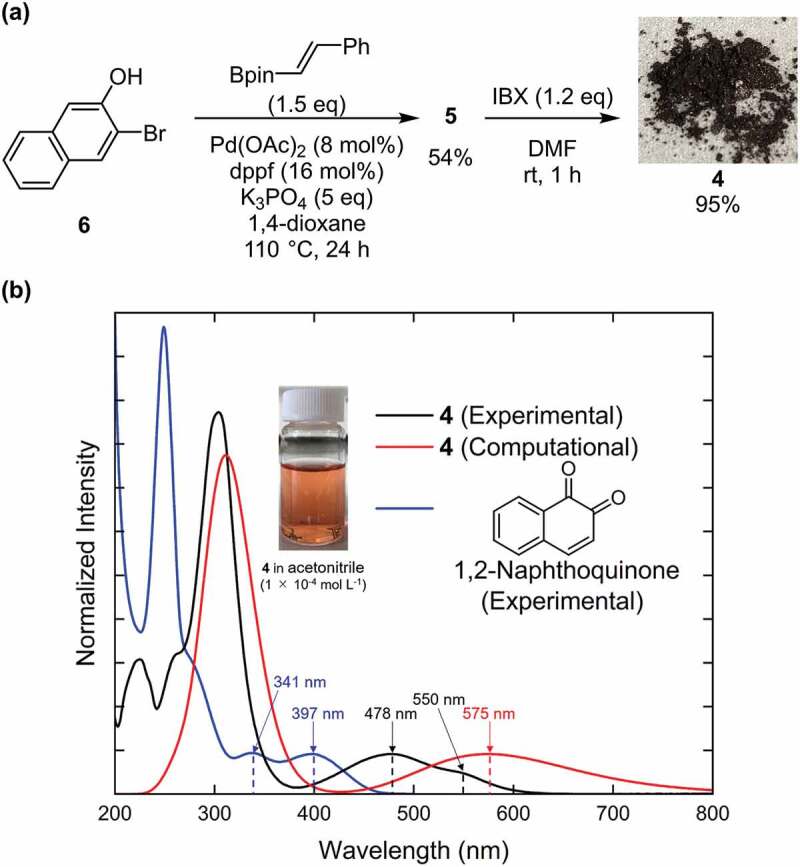
Synthetic route and UV-vis spectrum of molecule 4. (a) Synthesis process of 4. (b) the black and blue curves correspond to the UV-vis absorption spectra of the solution of 4 and that of 1,2-naphthoquinone in acetonitrile (1 × 10 ^−5^ mol L ^−1^), respectively. The red curve shows the computational absorption spectrum of 4 obtained by TD-DFT calculation at the APFD/6-31 G* level. Photograph of a solution of 4 in acetonitrile (1 × 10 ^−4^ mol L ^−1^) under ambient light is also shown.

The retrosynthetic analysis of **4** is shown in Figure 3(b). Compound **4** is prepared from 2-naphthol **5** by oxidation, which is synthesised from naphthol and an olefin through a cross-coupling reaction. As shown in Figure 4(a), using a commercially available compound (**6**) as the starting material, the Suzuki–Miyaura coupling reaction with vinylboronic acid pinacol ester gave **5**, which introduced the olefinic moiety into the naphthol skeleton [30]. Compound **5** was oxidised by 2-iodoxybenzoic acid (IBX) to form the target compound (**4**) as a dark-purple solid with 95% yield [31]. Compound **4** was found to be stable in air and soluble in many common solvents such as CHCl_3_, CH_2_Cl_2_, tetrahydrofuran, acetonitrile, and acetone. The product was characterised by NMR spectroscopy and mass spectrometry (see Figure S1-S4 of ESI).

The solution of **4** in acetonitrile (1 × 10^−5^ mol L^−1^) exhibited a red-purple color; its UV-vis absorption spectrum showed two peaks at 478 and 550 nm (Figure 4(b)). The corresponding two peaks of 1,2-naphthoquinone appear at 341 and 391 nm. This result shows that the substituent to 1,2-naphthoquinone in **4** contributes to red shift. The predicted result at the APFD/6-31 G* level agrees well with the experimental UV-vis absorption spectrum, where the former has a broad unimodal peak at 575 nm and the latter has the first peak at 550 nm. Although the 1,2-quinone derivatives are often unstable to air and light irradiation, we confirmed that the UV-vis spectrum of **4** did not change after its recrystallization as shown in Figure S12, hence **4** seems to be stable enough to apply as a dye.

## 4. Conclusion

We proposed CFGM in this paper and showed that it can elucidate strategic evolution of a DNMG and suggest an unconventional chromophore. In our experiments, ChemTS found light-absorbing molecules only from empirical observations without any knowledge of quantum chemical foundation. Early-day chemists in the 19^th^ century did not have such knowledge either. It is intriguing that the strategy learned by ChemTS is reminiscent of Armstrong’s quinoid theory [32] established in 1887. It states that only the compounds written in a quinoid form are coloured. Obviously, it is too simplistic and wrong in the eyes of current-day chemists. In the research of artificial intelligence, it is a widely asked question if AI ‘thinks’ like humans or how to make AI behave like humans [33]. The observation that ChemTS behaved like a chemist in the 19^th^ century is an encouraging sign for us. In future work, further integration of AI and quantum chemical knowledge may lead to a better system.

## Supplementary Material

Supplemental Material

## Data Availability

Chemical formulae of 1,2-naphthoquinone compounds generated by ChemTS, computational validation of the model molecules at a high level, and details of the chemical synthesis and characterisation of products are available in the Supplementary Information. The generated 45321 molecules are listed in the ALW_ChemTS/generated_mols/result.csv file. The file contains following information: generated molecules (SMILES), calculated absorption wavelengths and their oscillator strengths, and basic information such as molecular weight.

## Code availability

Our implementation of parallel version of ChemTS for fluorescent molecules is available at https://github.com/tsudalab/ALW_ChemTS.
