# Supplementary material for: Understanding the evolution of a de novo molecule generator via characteristic functional group monitoring
Source: Sci Technol Adv Mater. 2022 Jun 1;23(1):352–60. doi: 10.1080/14686996.2022.2075240 (PMC9176351; doi:10.1080/14686996.2022.2075240)
Supplement: Supplemental Material [file TSTA_A_2075240_SM8722.pdf]

Supplementary Information

for

Understanding the evolution of a de novo molecule generator via characteristic functional group monitoring

Takehiro Fujita[1,†], Kei Terayama[2,3,†], Masato Sumita[3,4,†,*], Ryo Tamura[3-6], Yasuyuki Nakamura[1], Masanobu Naito[1], Koji Tsuda[3,5,6,*]

1. *Data-driven Polymer Design Group, Research and Services Division of Materials Data and Integrated System (MaDIS), National Institute for Materials Science (NIMS), 1-2-1, Sengen, Tsukuba, Ibaraki 305–0047, Japan*

2. *Graduate School of Medical Life Science, Yokohama City University, Tsurumi-ku 230–0045, Japan*

3. *RIKEN Center for Advanced Intelligence Project, Tokyo 103–0027, Japan*

4. *International Center for Materials Nanoarchitectonics (WPI-MANA), National Institute for Materials Science (NIMS), Tsukuba 305–0044, Japan*

5. *Research and Services Division of Materials Data and Integrated System, National Institute for Materials Science (NIMS), Tsukuba 305-0044, Jpan*

6. *Graduate School of Frontier Sciences, The University of Tokyo, Kashiwa 277–8561, Japan*

[†]*These authors contributed equally.*

Correspondence and requests for materials should be addressed to M. S. (email: masato.sumita@riken.jp) or to K. Tsuda. (email: tsuda@k.u-tokyo.ac.jp)

1. Details of chemical synthesis

All reaction conditions dealing with oxygen and moisture sensitive compounds were carried out in a dry reaction vessel under nitrogen atmosphere. Unless otherwise noted, chemicals obtained from commercial supplies were used as received. Dehydrated 1,4-dioxane was degassed by nitrogen bubbling before using. ^{1}H NMR (400 MHz) and ^{13}C NMR (100 MHz) spectra were measured for a $CDCl_3$ solution of a sample and are reported in ppm (δ) from internal tetramethylsilane for ^{1}H NMR and from solvent peak for ^{13}C NMR. UV/vis spectrum was measured in CH_3CN at room temperature. Electrospray ionization mass (ESI-MS) spectrum was recorded on a spectrometer in the positive mode. A sample was injected as a CH_3CN solution.

1.2. Preparation of 5 and 4

Preparation of 5: A solution of 3-bromo-2-naphthol (1.00 g, 4.48 mmol), *trans*-2-phenylvinylboronic acid pinacol ester (1.60 g, 6.95 mmol), Pd $(OAc)_2$ (80.8 mg, 0.391 mmol), dppf (400 mg, 0.721 mmol), tripotassium phosphate (4.80 g, 22.6 mmol), and dehydrated 1,4-dioxane (20 mL) was heated at 110 °C for 24 h in a dry reaction vessel under nitrogen atmosphere. The reaction mixture was passed through a pad of silica gel with dichloromethane as the eluent and concentrated under reduced pressure to give a crude mixture. Purification by silica gel chromatography (dichloromethane/hexane = 8/2) afforded the title compound (602 mg, 54%). ^{1}H NMR ($CDCl_3$, 400 MHz): δ = 5.15 (s, 1H, -OH), 7.14 (s, 1H, Ar), 7.29-7.42 (m, 5H, -Ar), 7.32 (d, 1H, *J* = 16.5 Hz, -CH=CH-), 7.39 (s, 1H, Ar), 7.51 (d, 1H, *J* = 16.5 Hz, -CH=CH-), 7.59 (d, 1H, *J* = 7.3 Hz, -Ar), 7.59 (d, 1H, 8.2 Hz, -Ar), 7.65 (d, 1H, 8.2 Hz, -Ar), 7.79 (d, 1H, 7.3 Hz, -Ar), 8.01(s, 1H, -Ar); ^{13}C NMR ($CDCl_3$, 100 MHz): δ = 110.1, 123.4, 124.1, 126.0, 126.5, 126.7, 126.8, 127.0, 127.9, 128.0, 128.8, 129.3, 131.4, 134.0, 137.7, 151.7. ; HRMS (ESI) *m/z*: Calcd for $C_{18}H_{15}O_2$ $(M+H)^+$, 247.1123, found 247.1118.

Preparation of 4: A solution of **5** (200 mg, 0.812 mmol), IBX (purity >39%) (640 mg, 0.891 mmol), and DMF (5 mL) was stirred at room temperature for 1 h under an air. The reaction mixture was quenched with distilled water, and was extracted with ethyl acetate. The combined organic layers were washed with saturated aqueous NaCl solution, dried over $MgSO_4$, filtered and concentrated under reduced pressure to give a crude mixture. Purification by silica gel chromatography (dichloromethane/hexane = 9/1) afforded the title compound (201 mg, 95%). ^{1}H NMR ($CDCl_3$, 400 MHz): δ =7.05 (d, 1H, *J* = 16.5 Hz, -CH=CH-), 7.30 (m, 1H, -Ar), 7.37(dd, 1H, *J* = 7.6, 1.4 Hz, -Ar), 7.40 (m, 2H, -Ar), 7.45 (ddd, 1H, *J* = 7.6, 7.6, 1.4 Hz, -Ar), 7.47 (d, 1H, *J* = 16.5 Hz, -CH=CH-), 7.49 (s, 1H, -CH=), 7.54 (m, 2H, -Ar), 7.64 (ddd, 1H, *J* = 7.6, 7.6, 1.4 Hz, -Ar) 8.07 (dd, 1H, *J* = 7.6, 1.4 Hz, -Ar); ^{13}C NMR ($CDCl_3$, 100 MHz): δ = 121.1, 127.2, 128.8, 128.9, 130.0, 130.3, 130.6, 133.8, 135.1, 135.8, 136.2, 136.8, 138.8, 179.2, 180.6. ; HRMS (ESI) *m/z*: Calcd for $C_{18}H_{13}O_2$ $(M+H)^+$, 261.0915, found 261.0909.

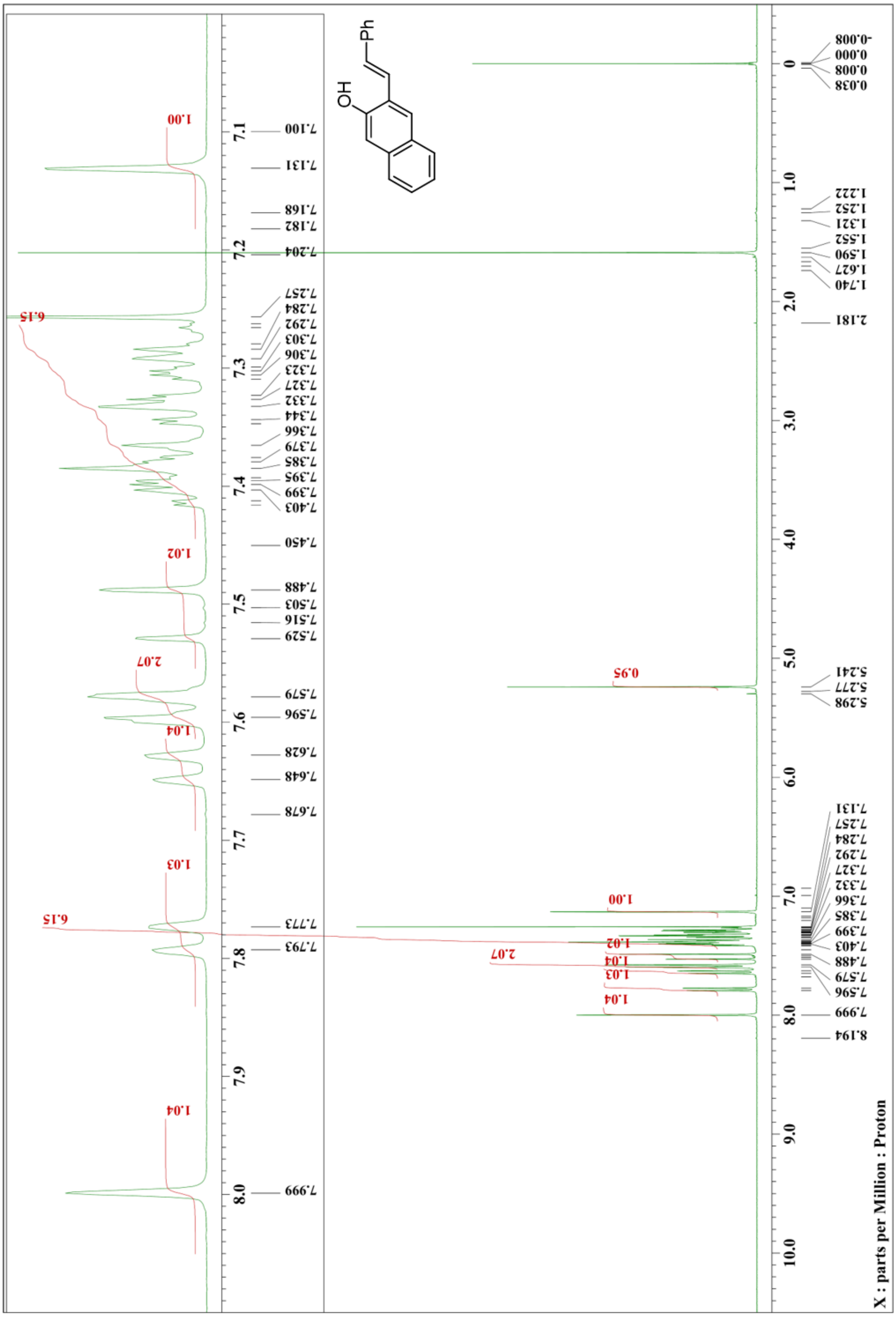

Figure S1. ^{1}H NMR spectrum of 5 in $CDCl_3$.

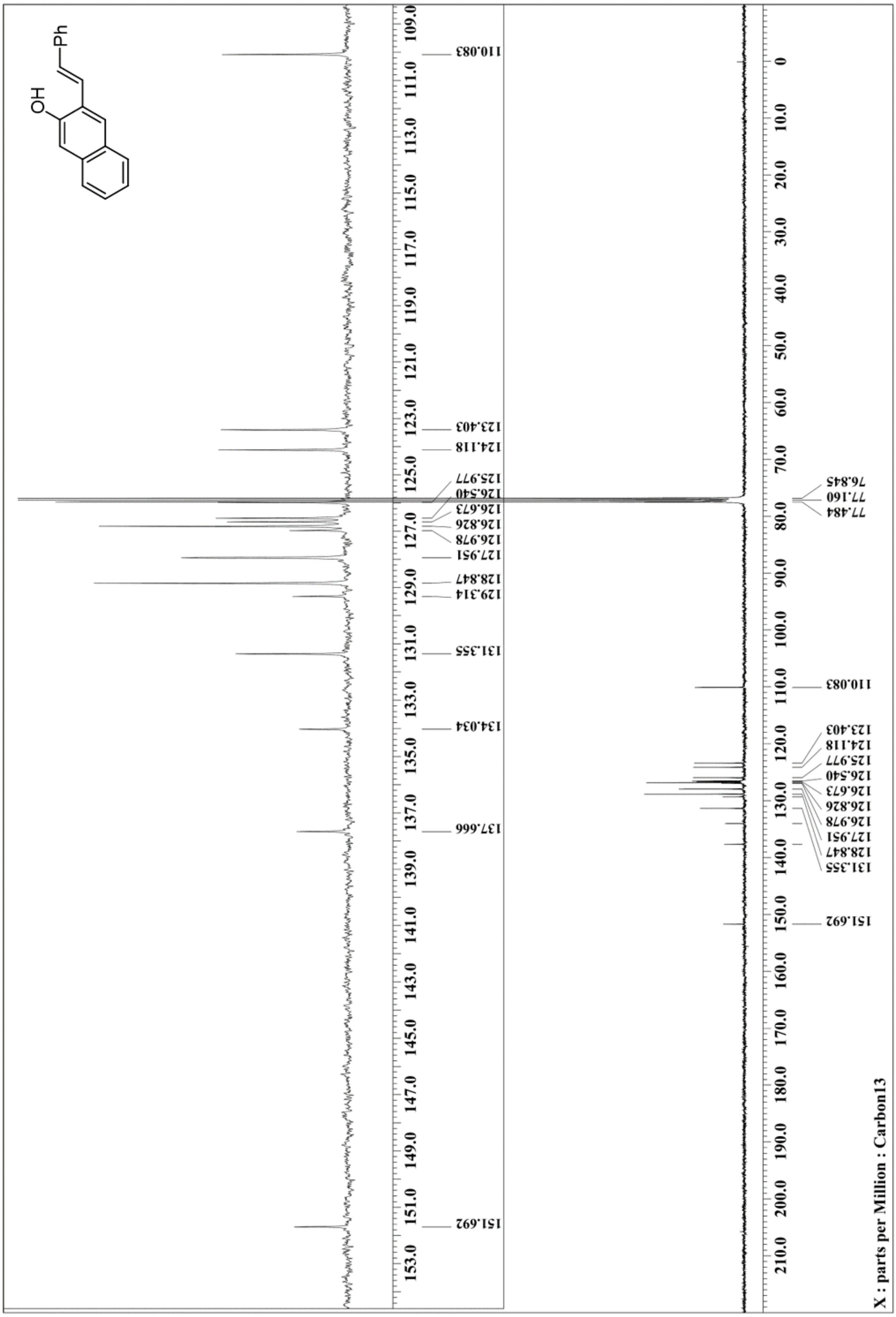

Figure S2. ^{13}C NMR spectrum of 5 in $CDCl_3$..

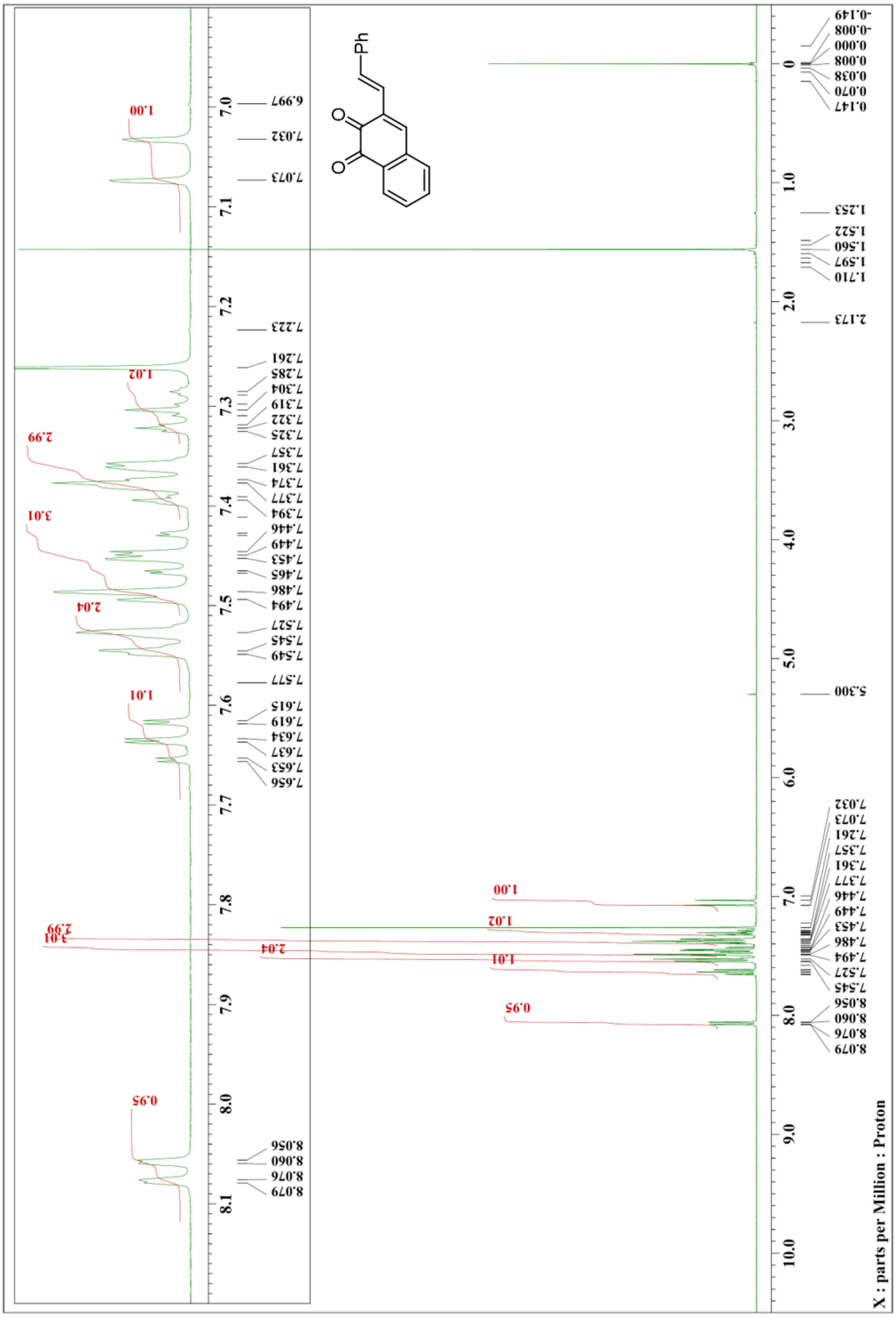

Figure S3. ^{1}**H NMR spectrum of 4 in** $CDCl_3$..

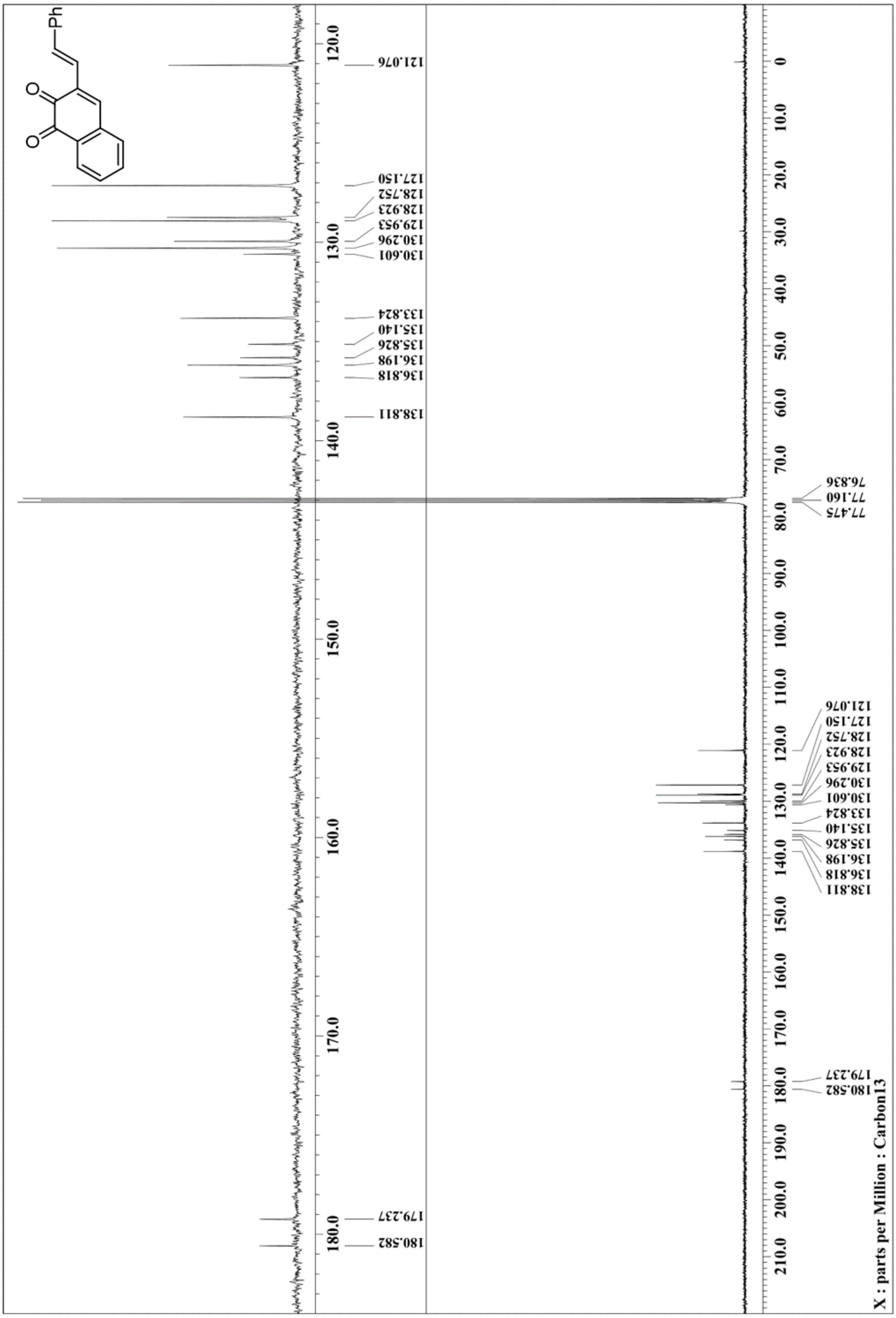

Figure S4. ^{13}C NMR spectrum of 4 in $CDCl_3$.

2. Dependence of MCTS parameter

To find the suitable MCTS parameter for molecule generation by ChemTS, we used ChemTS for generating long wavelength absorption molecules with MCTS parameters, C= 1, 2, and 4. In the case of C=1, the growth of averaged absorption wavelength is saturated around 800 nm after generating 25,000 molecules as shown in Fig. S5(a). On the other hand, in the case of C=4, the growth of averaged absorption wavelength is very slow as shown in Fig. 6(a) and the averaged absorption wavelength still 400 nm after generating 40,000 molecules. Hence, we concluded that C=2 was suitable parameter for designing long wavelength absorption molecule. Along with the absorption wavelength evolution, molecular properties (HOMO/LUMO gap and oscillator strength, molecular weight, conjugate length, and number of aromatic rings) in the case of C=1 shows the similar tendencies with the discussion in main text. In the case of C=4, the large change of molecular properties is not observed obviously. We thought that the computational time (120 h) is not enough to develop long wavelength absorption molecules with C=4.

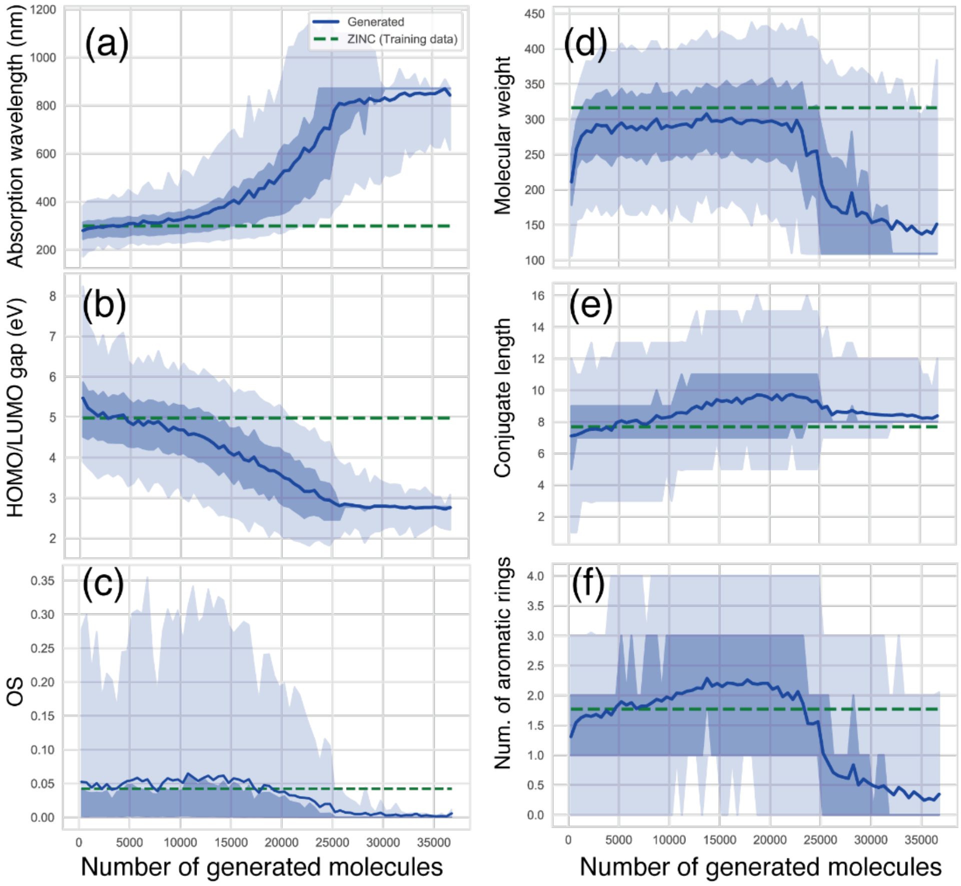

Figure S5. MCTS parameter, C = 1, evolution of several molecular properties. (a) absorption wavelength (nm) to S_1 excited state, (b) HOMO/LUMO gap (eV) (c) absorption intensity (oscillator strength; OS), (d) molecular weight (g mol^{-1}), (e) conjugate length, (f) number of aromatic rings as a

function of the increment of generated molecules. Average values of ZINC and generated molecules at each step are depicted by green broken line and blue solid line, respectively. The shaded area depicts the distribution profiles of generated molecules for each property. A thin shade area represents 5%–95% of the total distribution, while a dense shade area represents 15%–75% of the total distribution in each number of generated molecules.

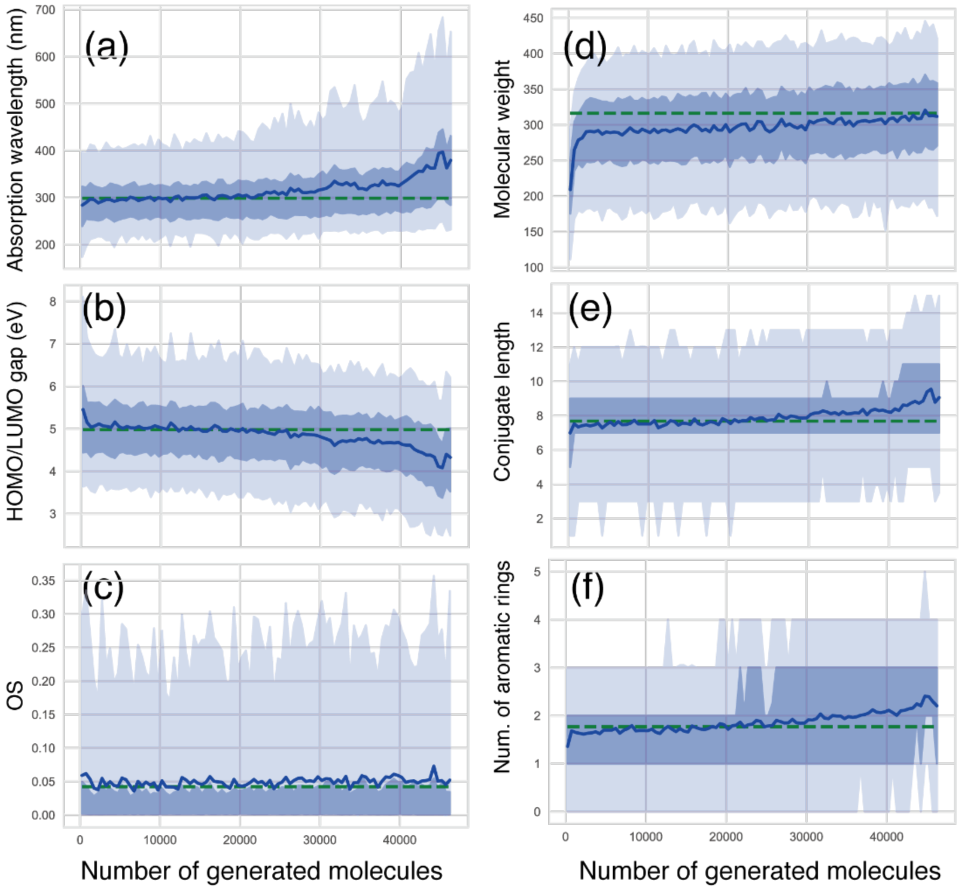

Figure S6. MCTS parameter, C = 4, evolution of several molecular properties. (a) absorption wavelength (nm) to S_1 excited state, (b) HOMO/LUMO gap (eV) (c) absorption intensity (oscillator strength; OS), (d) molecular weight (g mol^{-1}), (e) conjugate length, (f) number of aromatic rings as a function of the increment of generated molecules. Average values of ZINC and generated molecules at each step are depicted by green broken line and blue solid line, respectively. The shaded area depicts the distribution profiles of generated molecules for each property. A thin shade area represents 5%–95% of the total distribution, while a dense shade area represents 15%–75% of the total distribution in each number of generated molecules.

The dependence of MCTS parameter on the odds ratio is also investigated. In the case of C=1 and 4, although ChemTS still insisted on the ketone derivatives after 30,000 molecule generation, the odds ratios of 1,2-naphthoquinone derivatives grow after 20,000 molecule generation for C=1 and 30,000 molecule generation for C=4 as shown in Figure S7 and S8. Total odds ratios are summarised in Table S1 for each parameter. Except for C=4, 1,2-naphothoquinone derivatives shows high odds ratio. In the case of C=4, we thought that if more computational time had been available, ChemTS would have focused on the 1,2-naphotoquinone derivatives.

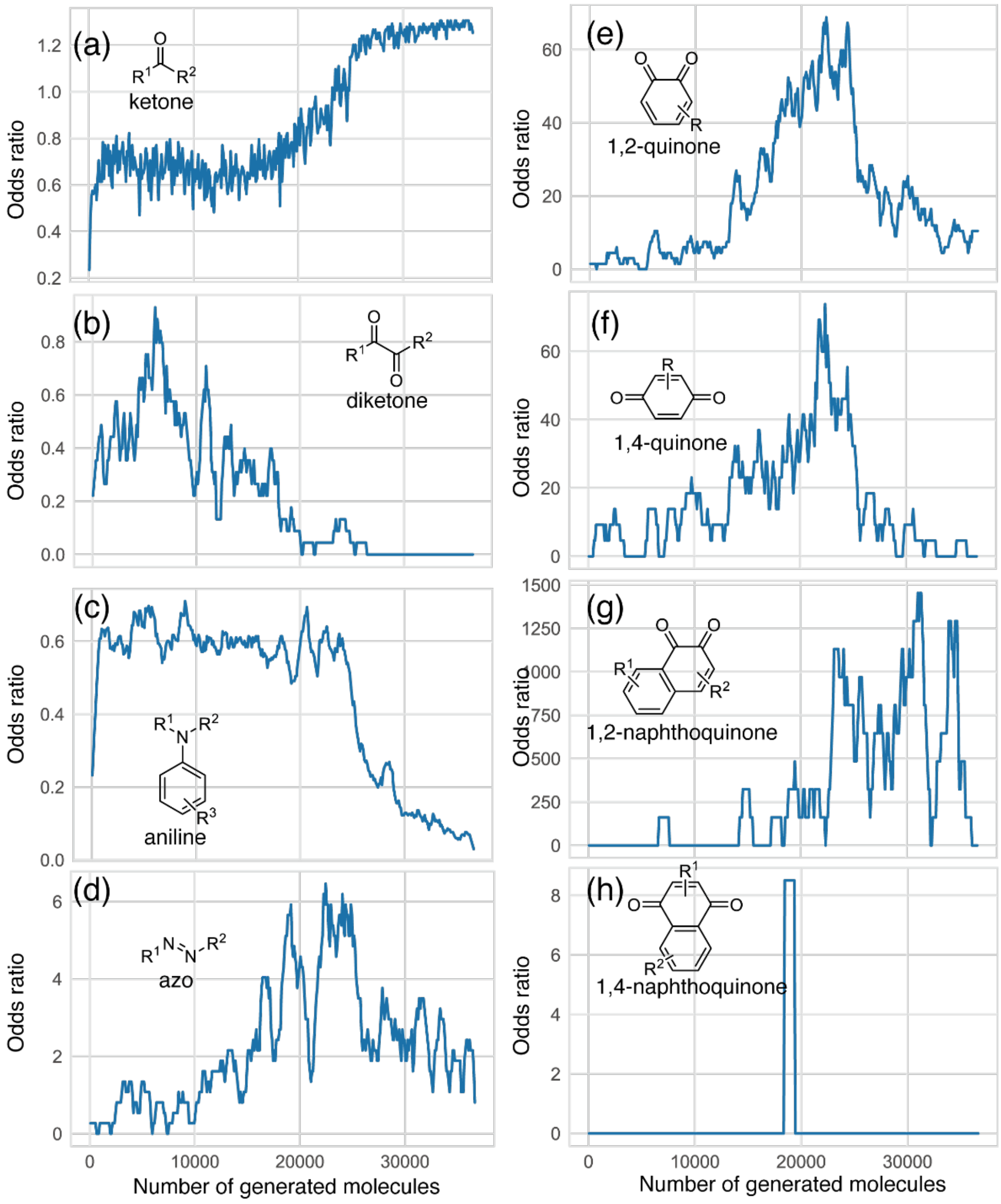

Figure S7. MCTS parameter, C = 1, odds ratio evolution of several functional groups shown in Table S1 as a function of the number of generated molecules. Odds ratios are computed for every

100 generated molecules.

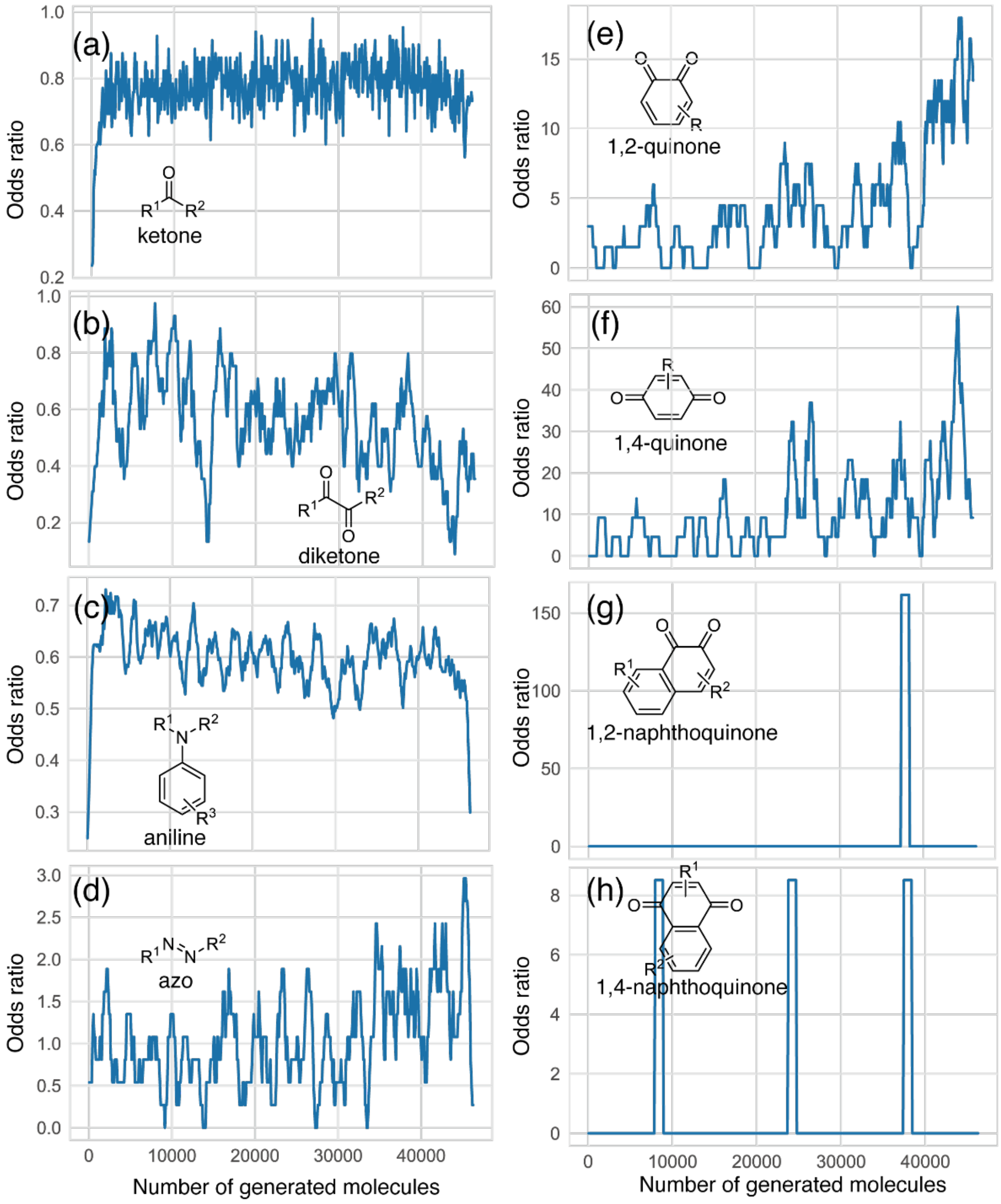

Figure S8. MCTS parameter, C = 4, odds ratio evolution of several functional groups shown in Table S1 as a function of the number of generated molecules. Odds ratios are computed for every 100 generated molecules.

Table S1. Total odds ratio (P_E) values of generated molecules by ChemTS with each parameter of MCTS.

Functional group	P_E (C=1)	P_E (C=2)	P_E (C=4)
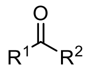 Ketone	0.891	0.649	0.773
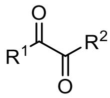 Diketone	0.229	0.375	0.558
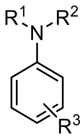 Aniline	0.455	0.537	0.602
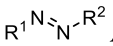 Azo	2.249	2.37	1.04
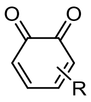 1,2-quinone	19.8	15.5	4.33
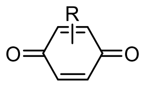 1,4-quinone	16.0	31.5	10.3
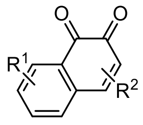 1,2-naphthoquinone	312	78.5	3.49
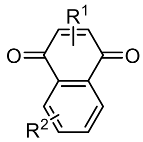 1,4-naphthoquinone	0.231	0.938	0.551

3. Generated molecules from Chem TS.

The 1,2-naphthoquinone derivatives generated by ChemTS (C=2) are shown in Fig. S9.

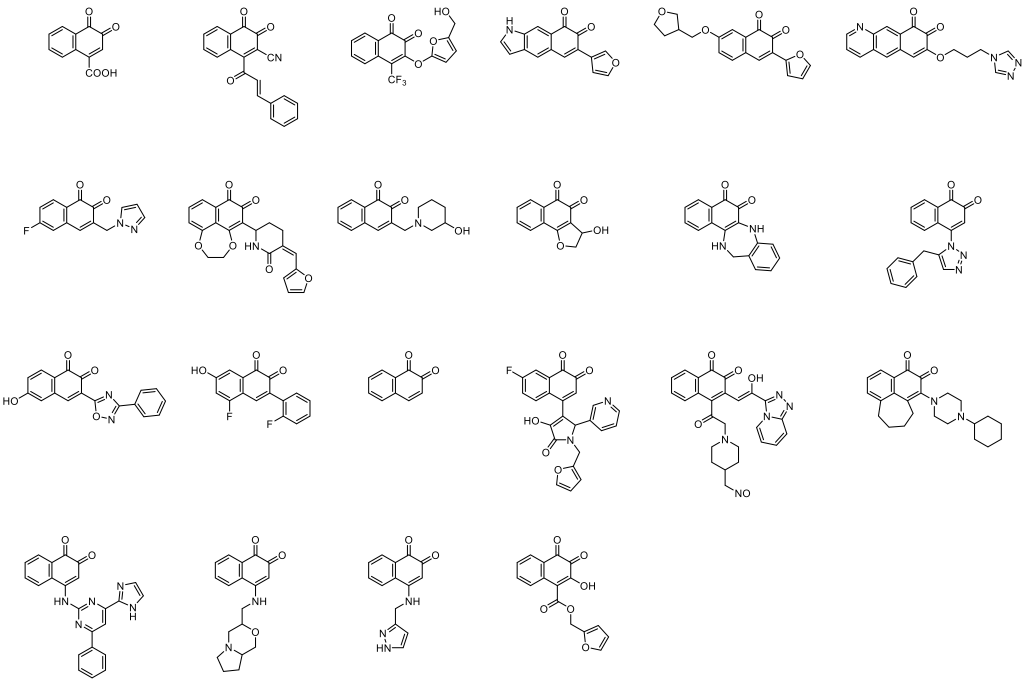

Figure S9. The generated 1,2-napthoquinone structures from Chem TS.

4. Computational studies of 1-4.

DFT calculations were carried using the Gaussian 16 program package.[1] The density functional theoretical (DFT) method was employed with the APFD hybrid functional[2] and the 6-31G(d) basis set.[3]

Table S2. Cartesian Coordinate of 1.

atom	x	y	z
O	2.386611	3.242562	0.959906
C	1.226141	3.006101	0.551089
C	0.775933	1.703046	0.015604
C	1.699393	0.614645	-0.176424
C	3.030725	0.438446	0.121809
O	3.903471	1.290457	0.705229
C	3.628955	-0.83862	-0.229113
N	2.979271	-1.862332	-0.800202
N	3.879581	-2.928786	-0.989749
C	5.069552	-2.509025	-0.516906

C	6.3199	-3.161446	-0.452449
C	7.39343	-2.499973	0.085293
C	7.252627	-1.165092	0.576388
C	6.051102	-0.522001	0.52121
N	4.965002	-1.189208	-0.021989
C	-0.595988	1.562041	-0.249763
C	-1.168291	0.202031	-0.495651
O	-0.579313	-0.691636	-1.135285
C	-2.47652	-0.167558	0.224626
N	-3.586147	-0.430547	-0.739137
C	-3.31375	-1.503473	-1.739788
C	-3.073435	-2.885227	-1.078297
C	-3.977123	-3.071403	0.176732
C	-4.316321	-4.544321	0.381007
N	-4.91022	-4.898823	1.762585
O	-4.911031	-3.913636	2.551753
C	-5.254125	-2.210559	0.042013
C	-4.900356	-0.696838	-0.052597
C	-1.515922	2.707588	-0.2833
C	-2.816913	2.594254	-0.820173
C	-3.660881	3.703602	-0.875982
C	-3.243667	4.956017	-0.41261
C	-1.957942	5.088847	0.09742
C	-1.098417	3.986144	0.151138
C	0.253095	4.195969	0.648212
O	0.671048	5.268123	1.107324
H	1.274385	-0.24152	-0.683677
H	3.418297	2.188708	0.90043
H	6.385311	-4.169942	-0.834471
H	8.359359	-2.985426	0.141987
H	8.103674	-0.649196	0.999372
H	5.855398	0.477789	0.86695
H	-2.794248	0.640507	0.883643
H	-2.225898	-1.045379	0.84142
H	-4.191366	-1.536711	-2.393951

H	-2.447977	-1.228376	-2.339822
H	-2.017082	-2.953988	-0.797703
H	-3.279813	-3.670264	-1.817636
H	-3.446941	-2.737421	1.074988
H	-3.429741	-5.19156	0.314622
H	-5.051681	-4.919234	-0.341847
H	-5.800076	-2.52804	-0.855465
H	-5.897152	-2.377729	0.910229
H	-4.844384	-0.268317	0.952904
H	-5.689372	-0.169613	-0.601227
H	-3.181845	1.632768	-1.174418
H	-4.654931	3.585632	-1.292145
H	-3.908231	5.809788	-0.459129
H	-1.581608	6.039265	0.456053

Table S3. Cartesian Coordinate of 2.

atom	x	y	z
C	5.909264	-1.855682	-0.000191
C	4.530987	-2.048449	-0.000224
C	3.657596	-0.951439	-0.000285
C	4.210053	0.346956	-0.00035
C	5.591746	0.532923	-0.000315
C	6.445355	-0.566609	-0.000229
H	1.887626	-2.182142	-0.000283
H	6.569883	-2.716004	-0.00013
H	4.123624	-3.054067	-0.000188
C	2.226959	-1.15016	-0.00027
C	3.300914	1.504735	-0.00051
H	5.979782	1.544837	-0.00037
H	7.51971	-0.422404	-0.000199
C	1.759789	1.227974	-0.000065
C	1.272418	-0.164643	-0.000181
O	3.671286	2.656423	-0.000373
O	1.037918	2.21324	0.000585

C	-0.124773	-0.54683	-0.000157
C	-1.291196	0.155931	0.000011
H	-0.286569	-1.618852	-0.000236
O	-1.459445	1.4914	0.000027
H	-0.576167	1.933241	-0.000348
C	-2.553169	-0.576416	0.00018
C	-4.680096	-1.130506	0.000378
C	-4.311058	1.26902	0.000055
C	-6.077377	-0.92796	0.000437
C	-5.658501	1.458532	0.000097
H	-3.574339	2.054168	-0.00008
C	-6.557893	0.352319	0.000293
H	-6.721453	-1.797774	0.000586
H	-6.038635	2.472246	-0.000019
H	-7.626145	0.534578	0.000326
N	-3.823792	-0.023088	0.000194
N	-2.663445	-1.898538	0.000332
N	-3.964406	-2.251873	0.000473

Table S4. Cartesian Coordinate of 3.

atom	x	y	z
C	4.919496	-2.14731	-0.167079
C	3.529028	-2.202848	-0.17522
C	2.767233	-1.02873	-0.087836
C	3.445264	0.204388	0.00936
C	4.838677	0.253105	0.019358
C	5.580284	-0.921206	-0.069991
H	0.887932	-2.078236	-0.159353
H	5.492149	-3.065988	-0.236616
H	3.025191	-3.161041	-0.249617
C	1.323285	-1.084891	-0.090218
C	2.65328	1.441589	0.103111
H	5.323749	1.219208	0.096452
H	6.663622	-0.883873	-0.064139

C	1.092179	1.321305	0.054537
C	0.467364	-0.016341	-0.013528
O	3.134752	2.545896	0.213462
O	0.473805	2.372711	0.068147
C	-0.96357	-0.26214	-0.022744
C	-2.067651	0.53757	0.053173
H	-1.196092	-1.315085	-0.125598
C	-3.430952	-0.040508	0.034811
C	-3.701442	-1.337133	0.494784
C	-4.494831	0.735246	-0.447608
C	-4.991692	-1.852452	0.44713
H	-2.907617	-1.937969	0.922851
C	-5.783765	0.215175	-0.500365
H	-4.297668	1.744296	-0.785257
C	-6.038163	-1.080832	-0.056413
H	-5.183336	-2.854354	0.816619
H	-6.592781	0.826004	-0.886895
H	-7.044761	-1.483732	-0.092418
O	-2.090977	1.87551	0.115307
H	-1.17516	2.233921	0.077073

Table S5. Cartesian Coordinate of 4.

atom	x	y	z
C	4.974918	-1.90424	0.000155
C	3.59303	-2.070375	-0.00017
C	2.741677	-0.957446	-0.000176
C	3.314191	0.331315	0.000069
C	4.698919	0.490344	0.000403
C	5.532347	-0.624803	0.000455
H	0.94478	-2.155057	-0.000464
H	5.620201	-2.77614	0.000163
H	3.166667	-3.068325	-0.000405
C	1.303211	-1.128297	-0.000297
C	2.426015	1.513523	-0.00007

H	5.105038	1.495137	0.000591
H	6.609255	-0.499548	0.000737
C	0.876762	1.27627	-0.00022
C	0.379013	-0.125554	-0.000161
O	2.836111	2.651771	-0.000095
O	0.16131	2.252803	-0.000424
C	-1.036051	-0.456996	-0.000076
C	-2.086486	0.388976	-0.000066
H	-1.227222	-1.528259	0.000051
H	-1.889872	1.453127	-0.000145
C	-3.497736	0.007228	0.000048
C	-3.949948	-1.324427	-0.000135
C	-4.462785	1.028334	0.000262
C	-5.306805	-1.616835	-0.000046
H	-3.23723	-2.141482	-0.000379
C	-5.822863	0.736512	0.000353
H	-4.133248	2.062551	0.000359
C	-6.251737	-0.588482	0.000185
H	-5.633079	-2.651725	-0.000197
H	-6.54745	1.543954	0.000553
H	-7.311235	-0.821118	0.000213

Table S6. TD-DFT vertical one-electron excitations of 1-4 calculated at the APFD/6-31G* level of theory.

Molecule	Energy (eV)	Wavelength (nm)	Oscillator strength (*f*)	Contributions
**1**	1.3071	948.55	0.1184	HOMO → LUMO (0.70445)
	2.4312	509.97	0.0194	HOMO-3 → LUMO (0.69501)
	3.0418	407.6	0.1592	HOMO-8 → LUMO (0.15733)
				HOMO-7 → LUMO+1 (-0.26341)
				HOMO-6 → LUMO+2 (0.31228)
				HOMO-5 → LUMO+3 (0.16229)
				HOMO → LUMO+2 (0.50583)
**2**	1.5122	819.87	0.0946	HOMO → LUMO (0.70229)

	3.4412	360.29	0.5622	HOMO-5 → LUMO (0.30817)
				HOMO → LUMO+1 (0.61870)
	3.7376	331.72	0.6404	HOMO-5 → LUMO (0.54147)
				HOMO → LUMO+1 (-0.31018)
				HOMO → LUMO+2 (0.21627)
				HOMO → LUMO+3 (-0.17581)
**3**	1.6376	757.11	0.0803	HOMO → LUMO (0.70342)
	3.5643	347.85	0.1353	HOMO-6 → LUMO (-0.29759)
				HOMO-4 → LUMO (0.43881)
				HOMO → LUMO+1 (-0.42843)
				HOMO → LUMO+2 (0.10143)
	3.5791	346.41	0.0541	HOMO-6 → LUMO (0.59399)
				HOMO-4 → LUMO+1 (0.18294)
				HOMO → LUMO+1 (-0.25880)
	3.7360	331.86	0.0251	HOMO-5 → LUMO+1 (0.67554)
				HOMO-2 → LUMO+1 (0.10776)
	3.8781	319.7	0.7502	HOMO-4 → LUMO (0.47965)
				HOMO → LUMO+1 (0.48032)
				HOMO → LUMO+2 (0.11425)
	4.2293	293.15	0.1383	HOMO-4 → LUMO (-0.14889)
				HOMO → LUMO+2 (0.66847)
**4**	2.1579	574.56	0.1343	HOMO → LUMO (0.69944)
	3.7627	329.50	0.2781	HOMO-4 → LUMO (0.48108)
				HOMO → LUMO+1 (0.49246)
				HOMO → LUMO+2 (0.10714)
	4.0247	308.06	0.9317	HOMO-4 → LUMO (-0.46977)
				HOMO → LUMO+1 (0.49246)
				HOMO → LUMO+2 (-0.10500)
	4.831	269.39	0.9317	HOMO-5 → LUMO (-0.10028)
				HOMO-1 → LUMO+1 (0.68444)
	4.6211	268.3	0.1050	HOMO-5 → LUMO (-0.12278)
				HOMO-3 → LUMO+1 (-0.11199)
				HOMO-2 → LUMO+1 (-0.11663)
				HOMO → LUMO+2 (0.64519)
				HOMO → LUMO+4 (0.12143)

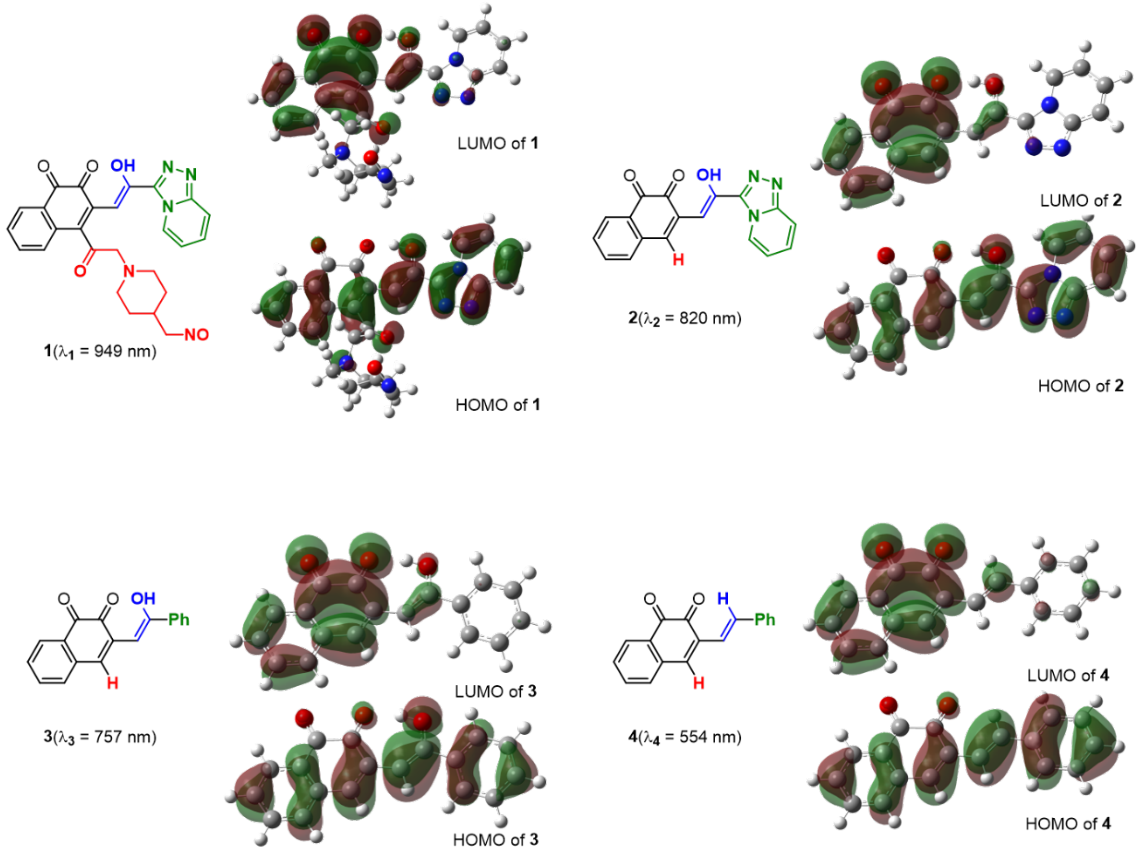

Figure S10. The HOMO-LUMO orbitals of 1-4 computed at the APFD/6-31G* level of theory. Surfaces are drawn at an isodensity value of 0.02.

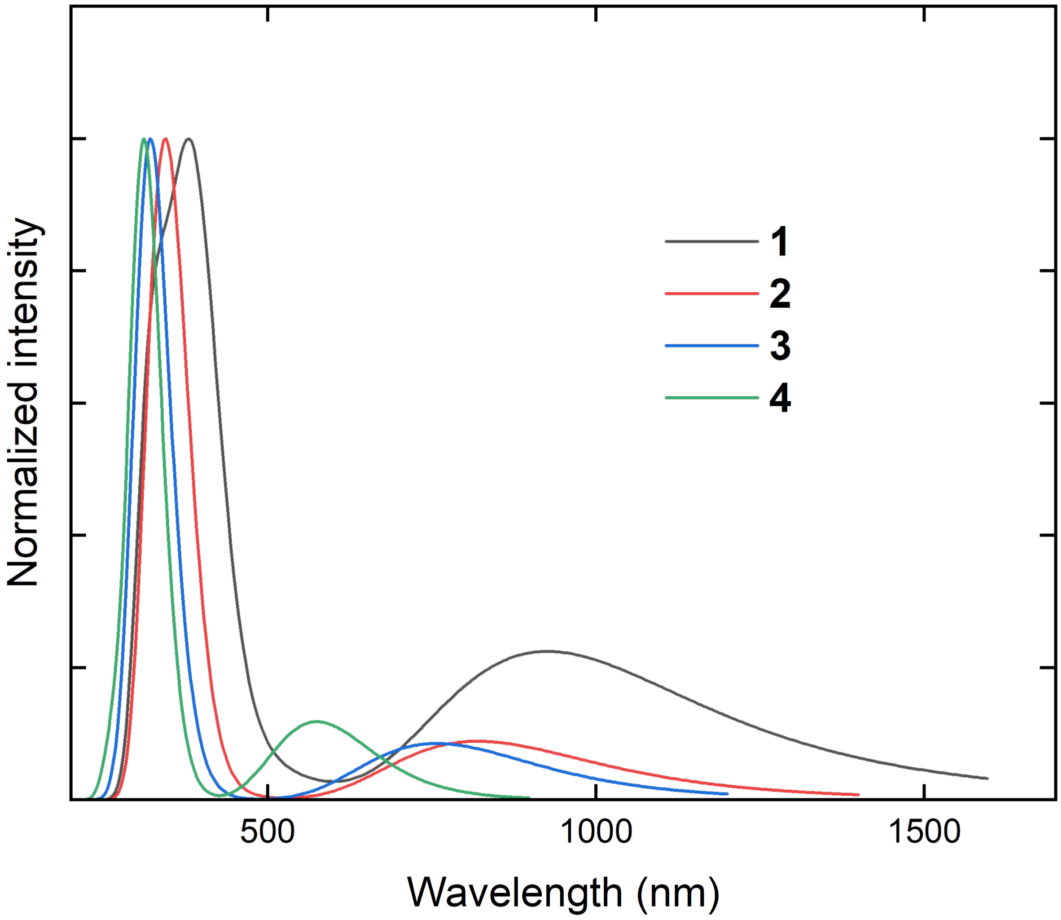

Figure S11. Computational spectra of 1-4 obtained by TD-DFT calculation at the APFD/6-31G* level of theory.

4. Recrystallization of 4.

To examine the effects of trace impurities, **4** was further purified by recrystallization after refining by silica gel chromatography. Hexane/CH_2Cl_2 was used as the recrystallization solvent. The spectra of UV-vis in acetonitrile (Figure S12) and ^{1}H NMR in $CDCl_3$ (Figure S13) were measured. In each measurement, there was no change in the spectra before and after recrystallization. These results indicate that **4** was sufficiently purified by silica gel chromatography.

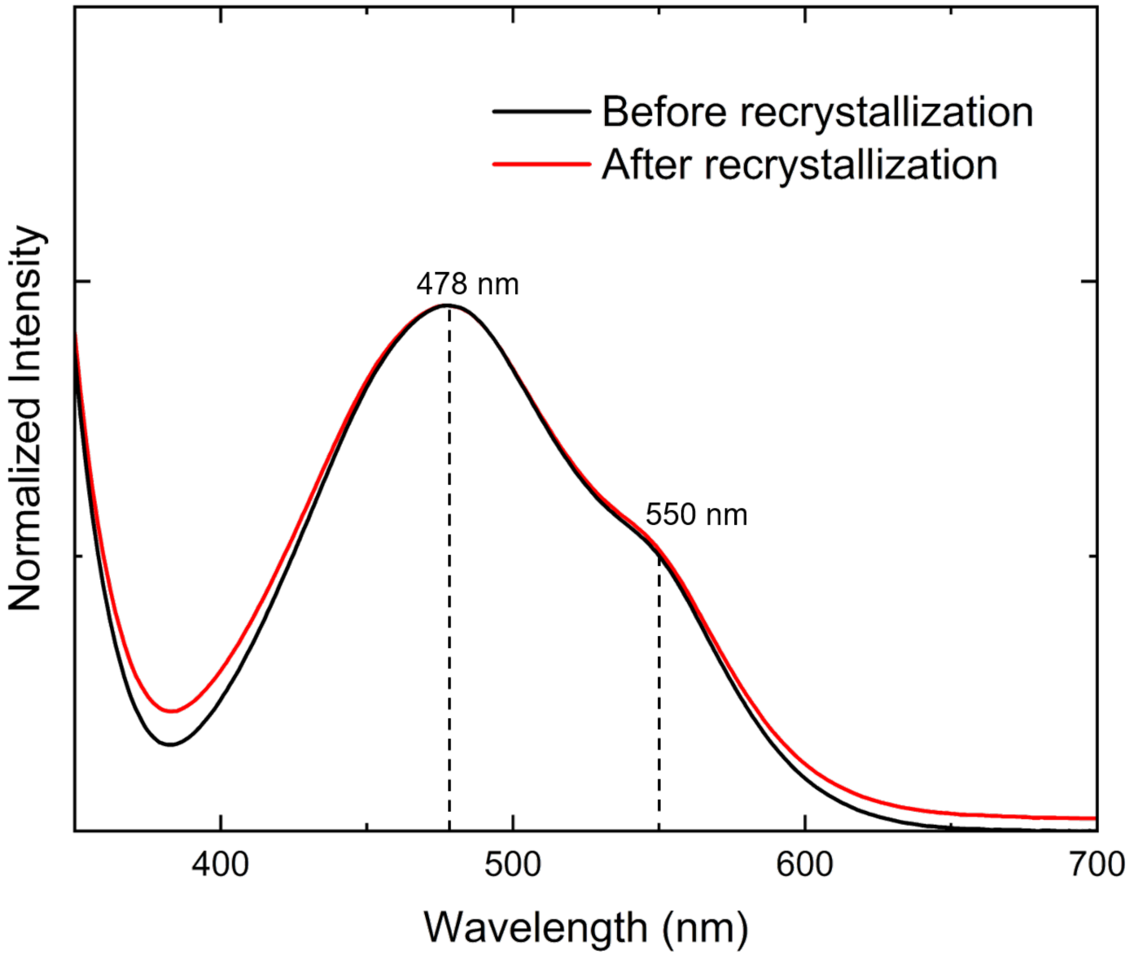

Figure S12. UV-vis absorption spectra of **4** in acetonitrile before and after recrystallization.

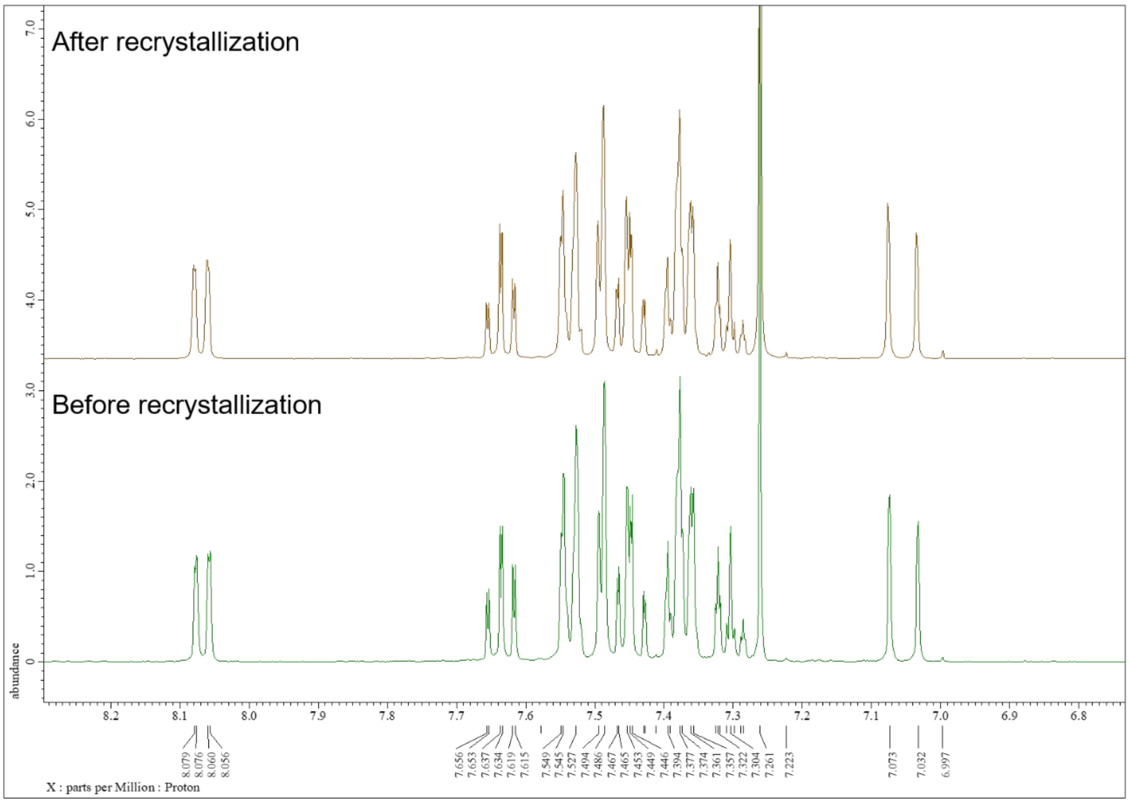

Figure S13. ^{1}H NMR spectra of **4** in $CDCl_3$ before and after recrystallization.

References

1. Frisch, M. J. *et al.*, Gaussian 16, Revision B.01, Gaussian, Inc., Wallingford CT, 2016.
2. Austin, A., Petersson, G., Frisch, M. J., Dobek, F. J., Scalmani, G. and Throssell, K. *J. Chem. Theory Comput.* **8**, 4989−5007(2012).
3. Hehre, W. J., Radom, L., Schleyer, P. v. R.,and Pople, J. A. *Ab initio Molecular Orbital Theory*. (John Wiley, 1986).
